# Kinship Modulates Carbon Allocation and Phosphorus Acquisition in Chinese Fir–AMF Networks Under Neighbor P Limitation

**DOI:** 10.3390/plants15050703

**Published:** 2026-02-26

**Authors:** Zihao Zhao, Hongjian Wei, Hui Hu, Yuxin Yao, Jing Liang, Pengfei Wu

**Affiliations:** 1 College of Forestry, Fujian Agriculture and Forestry University, Fuzhou 350002, China; 18159086785@163.com (Z.Z.); weihongjian@fafu.edu.cn (H.W.); huhui5258548@163.com (H.H.); yyx20010912@163.com (Y.Y.); fjyangjoung@126.com (J.L.); 2 Chinese Fir Engineering Technology Research Center of the State Forestry and Grassland Administration, Fuzhou 350002, China; 3 Fujian Provincial Colleges and University Engineering Research Center of Plantation Sustainable Management, Fuzhou 350002, China

**Keywords:** arbuscular mycorrhizal fungi, Chinese fir, phosphorus limitation, carbon–phosphorus trade, kinship

## Abstract

Phosphorus (P) deficiency in forest soils is a key constraint on the sustainable management and productivity of Chinese fir (*Cunninghamia lanceolata*) plantations. This study investigated how P limitation alters the reciprocal exchange of “photosynthetic carbon and mineral phosphorus” between Chinese fir and arbuscular mycorrhizal fungi (AMF) when the focal plant grows adjacent to neighbors with different degrees of relatedness. An indoor pot experiment simulating heterogeneous P supply was conducted using clonal seedlings of Chinese fir No. 36 as the focal plant, with Chinese fir No. 36, Chinese fir No. 41, and *Schima superba* as neighboring plants to establish three two-plant combinations: a kin pair (No. 36 + No. 36), a close-kin pair (No. 36 + No. 41), and an unrelated-kin pair (No. 36 + *S. superba*). *Funneliformis mosseae* was inoculated into the shared root-zone room connecting the two plants, and the neighbor was subjected to a gradient of P limitation (sufficient P, low P, and zero P). Meanwhile, the focal No. 36 plant received ^13^CO_2_ pulse labeling to form a “Chinese fir–AMF–P-limited neighbor” symbiotic network in which No. 36 served as the ^13^C donor. AMF colonization, seedling growth, and changes in ^13^C enrichment and P concentration in plant tissues of the focal plant were quantified. Neighbor P limitation significantly increased AMF colonization in roots and whole-plant P concentration of the focal Chinese fir. Following ^13^CO_2_ pulse labeling, whole-plant ^13^C enrichment of the focal plant increased significantly under the neighbor zero P treatment, suggesting enhanced carbon allocation under severe neighbor P limitation. Moreover, under the neighbor zero P treatment, focal plants grown with an unrelated-kin neighbor showed significant increases in stem P concentration (1.86 g·kg^−1^) and stem atom% ^13^C (1.50%), whereas focal plants grown with a kin neighbor exhibited a significant increase in root Atom% ^13^C (1.29%). These patterns indicate that neighbor relatedness may modulate carbon allocation and P acquisition within the mycorrhizal network: in the kin context, the focal plant tended to allocate more photosynthetic carbon belowground and may partially subsidize the AMF carbon demand (i.e., a higher C reward), coinciding with a relatively weaker P accumulation in its own tissues; in contrast, in the unrelated kin context, carbon allocation shifted toward stems and was associated with strengthened P accumulation in stem tissues. Overall, the results highlight the dynamic nature of AMF-mediated carbon–nutrient reciprocity across hosts of contrasting relatedness and provide new insights into how mycorrhizal networks may facilitate plant adaptation to nutrient limitation.

## 1. Introduction

Phosphorus (P) is an essential nutrient for plant growth and development and plays pivotal roles in multiple metabolic processes and the biosynthesis of key cellular constituents [1]. Although soils generally contain substantial amounts of total P, most P occurs in organic forms or as mineral-bound salts, which are characterized by low mobility and strong spatial heterogeneity. Consequently, the pool of plant-available P is often limited [2], and soil P deficiency constrains more than 30% of global agricultural and forestry production [3]. Arbuscular mycorrhizal fungi (AMF) are among the most widespread and intimate soil fungal symbionts of plants and are central to plant nutrient acquisition [4]. After symbiosis is established, AMF develop extraradical hyphae that extend beyond the root depletion zone, thereby enlarging the effective soil volume explored for P and transporting absorbed P to cortical cells in the host root, which helps alleviate P limitation [5]. In return, the host allocates photosynthetically derived carbohydrates and lipids to the extraradical hyphae as a “C reward”, providing energy and substrates for AMF growth, hyphal development, and reproduction, thereby forming a reciprocal exchange based on photosynthetic C and mineral nutrients [6].

In natural ecosystems, arbuscular mycorrhizal fungi (AMF) often colonize multiple host plants simultaneously. Through hyphal interconnections and anastomosis, AMF form structurally complex arbuscular mycorrhizal networks (AMNs) [7], which function as important pathways for resource exchange and regulation in soils. These networks can facilitate the transfer and redistribution of limited pools of readily available P and, to some extent, carbon (C) sources within the network, thereby extending plant–fungus interactions from a single plant “root–fungus” association to functional coupling among multiple plants and enhancing tolerance to P limitation [8,9]. However, under P deficiency, plant growth and metabolism are frequently suppressed, which can reduce photosynthesis and consequently decrease the fixation of photosynthetic C; accordingly, the C supply available to AMF from host plants also declines [10,11]. Given that a primary ecological payoff of AMF symbiosis is the acquisition of sufficient photosynthates from hosts to support spore production and hyphal development [12], an unresolved question is whether AMF alter the efficiency and/or allocation of nutrient transfer through hyphae among different hosts when the “C reward” provided by hosts is highly asymmetric under nutrient-stressed conditions.

Some scholars propose that, within natural plant communities, larger individuals or healthier plants possess greater effective photosynthetic surface area, and AMF therefore preferentially allocate mineral nutrients through arbuscular mycorrhizal networks (AMNs) to hosts that can offer more photosynthetic C in exchange, thereby securing a higher “C reward” [13,14]. In a *Medicago truncatula* system, Fellbaum et al. [15] observed that AMF consistently allocated a higher proportion of P to unrelated shaded hosts with stronger photosynthetic capacity, whereas shaded hosts with constrained photosynthesis were more likely to become disadvantaged recipients of mineral nutrients within AMNs. In contrast, other studies suggest that AMF can enhance community-level nutrient use efficiency and promote plant diversity by supplying mineral nutrients to competitively weaker individuals [16,17]. For example, Walder et al. [18] reported that a weakly competitive flax (*Linum usitatissimum*) contributed very little carbon to AMF yet received substantial supplementation of P, N, and other nutrients, resulting in a marked growth benefit. Collectively, these findings indicate that AMF resource allocation is rarely static or evenly distributed; rather, it can be dynamically regulated by host C-supply capacity, the intensity of nutrient demand, and external environmental constraints, thereby reshaping the “carbon–mineral nutrient” trade within AMNs and ultimately altering competitive interactions among plants [19,20]. Therefore, elucidating how P limitation modulates the reciprocal exchange of “photosynthetic C and mineral nutrients” among different hosts is critical for maintaining the stability of plant communities.

An issue that warrants further investigation is that neighboring plants within a community may not only recognize kin via specific leaf volatiles, photoreceptor-mediated cues, and root exudates, but also establish kin-recognition networks through soil microorganisms such as AMF and rhizobia, thereby mitigating resource competition between neighbors [21]. Close to animals, plants can exhibit kin recognition and kin-biased responses during growth and development: individuals assess the relatedness of surrounding plants and adjust their competitive strategies accordingly [22]. For instance, *Cakile edentula* markedly suppresses root growth when growing adjacent to closely related neighbors [23]. In *Pseudotsuga menziesii* and other species, encounters with kin neighbors have been associated with increased AM colonization accompanied by reduced investment in root growth, which may help avoid overexploitation of shared belowground resources and relieve intraspecific competitive pressure [24,25]. Booth et al. [26] further demonstrated that AMF can transport carbon acquired from adult trees through AMNs to related individuals, thereby reducing intraspecific competition and enhancing population stability. Together, these observations suggest that plant roots can deploy kin-dependent competitive strategies, and that cooperation among closely related individuals may increase inclusive fitness, benefiting both individuals and populations—an advantage that can be critical for maintaining competitiveness under changing environments [27]. Accordingly, some plants may initiate kin-recognition behaviors to avoid maladaptive escalation of competition, and such recognition may be particularly pronounced among genotypes with high relatedness. Therefore, examining how kin recognition modulates the provision of “C reward” from plants to AMF offers a new perspective for mechanistic studies on the reciprocal exchange of “photosynthetic C and mineral nutrients” between AMF and plants.

Chinese fir (*Cunninghamia lanceolata*) is the most important timber and plantation tree species in subtropical southern China. It is distributed across 17 provinces and administrative regions, with a plantation area of approximately 9.902 million ha [28]. As a lineage proposed to have originated in the Late Jurassic of the Mesozoic [29], Chinese fir has likely undergone long-term coevolution with AMF, and extensive belowground mycorrhizal hyphal networks may occur in the interlaced root zone, facilitating mutual dependence among neighboring individuals [30]. Previous studies have shown pronounced differences among Chinese fir clonal plantations derived from distinct geographic provenances in their capacity and efficiency to recruit AMF in soil [31]. Moreover, P limitation can induce Chinese fir roots to release L-arginine and volatile organic compounds (VOCs) such as n-decane, which may strengthen kin-recognition capacity [32,33]. Collectively, these characteristics make Chinese fir a suitable system to investigate AMF-mediated kin recognition among neighboring plants.

Thus, in this study, we constructed a “focal Chinese fir–AMF–P-limited neighbor” symbiotic network system with varying genetic relatedness. Combined with ^13^CO_2_ pulse labeling of the focal Chinese fir, we quantified AMF colonization, seedling growth, and the dynamics of ^13^C enrichment and P concentration in tissues of the focal Chinese fir to test the following hypotheses: (1) Chinese fir copes with the P-limited environment of neighboring plants by regulating its mutualistic relationship with AMF. (2) Kin recognition regulates the strategies employed by Chinese fir in response to phosphorus limitation in neighboring plants.

## 2. Results

### 2.1. Effects of Neighboring Plant P Limitation on AMF Colonization Rate in Chinese Fir Roots

**Figure 1:**
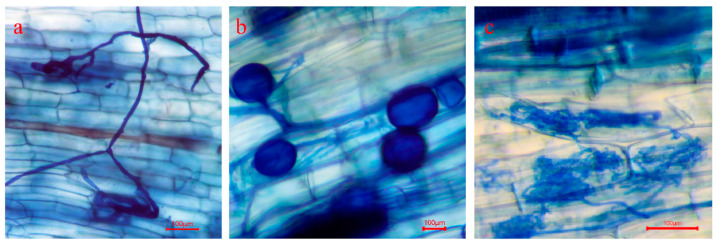
Typical AMF structures in Chinese fir roots: (**a**) hyphae, (**b**) vesicles, and (**c**) arbuscules.

**Figure 2:**
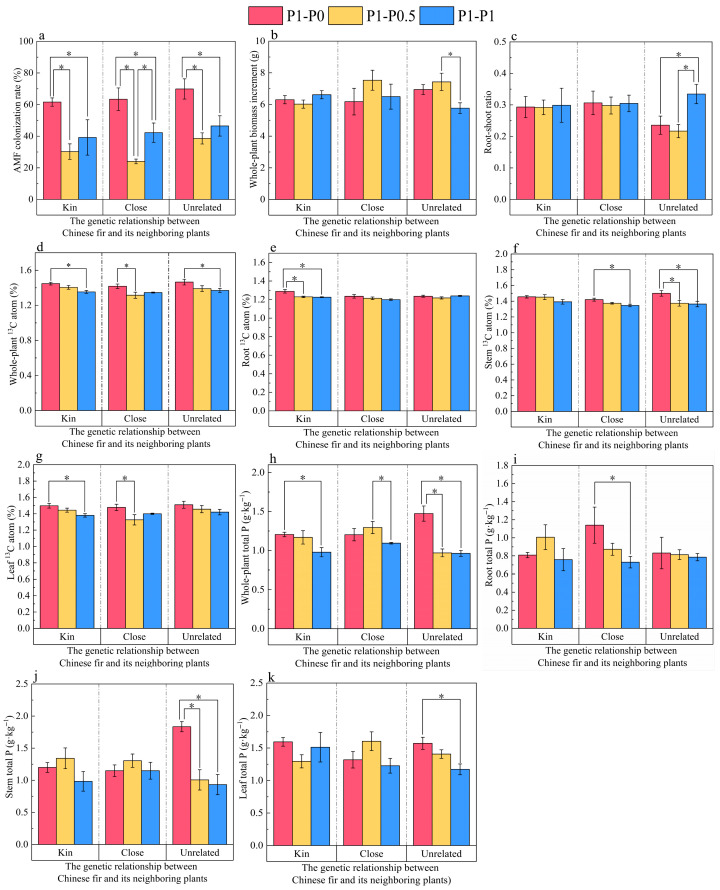
Responses of the focal Chinese fir (clone No. 36) to neighbor P supply gradients under different levels of neighbor relatedness. (**a**) AMF colonization rate. (**b**) Whole-plant dry mass biomass increment. (**c**) Root-to-shoot ratio. (**d**) Whole-plant ^13^C abundance (atom% ^13^C). (**e**–**g**) ^13^C abundance (atom% ^13^C) in roots (**e**), stems (**f**), and leaves (**g**). (**h**–**k**) Total P concentration in the whole plant (**h**), roots (**i**), stems (**j**), and leaves (**k**). Kin, Close, and Unrelated indicate that the focal Chinese fir was grown adjacent to kin, Close, or unrelated neighbor plant, respectively. P1–P0, P1–P0.5, and P1–P1 denote neighbor P supply levels of zero P, low P, and P sufficiency, respectively. Data are presented as mean ± SE (*n* = 5). Asterisks indicate significant differences among treatments (*p* < 0.05, LSD test).

*Funneliformis mosseae* formed mycorrhizae with the roots of Chinese fir, and typical mycorrhizal structures were developed inside the roots (Figure 1). Across the three relatedness combinations (Kin, Close, and Unrelated), AMF colonization in roots of the focal Chinese fir was significantly higher under the neighbor zero P treatment (P1–P0) than under the neighbor P-supplied treatments (P1–P0.5 and P1–P1) (*p* < 0.05) (Figure 2a).

### 2.2. Effects of Neighboring Plant P Limitation on the Growth of Chinese Fir

In the Unrelated combination, the whole-plant biomass increment of the focal Chinese fir under neighbor low P (P1–P0.5) was significantly higher than that under neighbor P sufficiency (P1–P1) (*p* < 0.05). In the Kin and Close combinations, whole-plant biomass increment did not differ significantly among neighbor P treatments (*p* > 0.05) (Figure 2b). In the Unrelated combination, the root-to-shoot ratio of the focal Chinese fir under neighbor zero P (P1–P0) was significantly reduced by 29.56% relative to P1–P1 (LSD; *p* < 0.05), whereas no significant difference was observed between P1–P0 and P1–P0.5 (*p* > 0.05). No significant effects of neighbor P supply on the root-to-shoot ratio were detected in the Kin or Close combinations (*p* > 0.05) (Figure 2c).

### 2.3. Effects of Neighboring Plant P Limitation on ^13^C Abundance in Different Organs of Chinese Fir

The responses of ^13^C abundance (atom% ^13^C) in the whole plant and individual organs of the focal Chinese fir differed among neighbor P treatments and relatedness combinations. At the whole-plant level, across the Kin, Close, and Unrelated combinations, whole-plant atom% ^13^C under the neighbor zero P treatment (P1–P0) was significantly higher than that under either neighbor P sufficiency (P1–P1) or neighbor low P (P1–P0.5) (*p* < 0.05) (Figure 2d). At the root level, a significant effect was detected only in the Kin combination, where root atom% ^13^C under P1–P0 was higher than that under P1–P0.5 and P1–P1 (*p* < 0.05) (Figure 2e). At the stem level, stem atom% ^13^C in the Close combination under P1–P0 was 5.50% higher than P1–P1 (*p* < 0.05); in the Unrelated combination, P1–P0 was significantly higher than P1–P0.5 and P1–P1 (*p* < 0.05) (Figure 2f). At the leaf level, leaf atom% ^13^C in the Kin combination was 8.55% higher under P1–P0 than under P1–P1 (*p* < 0.05); in the Close combination, P1–P0 was significantly higher than P1–P0.5 (*p* < 0.05) (Figure 2g).

### 2.4. Effects of Neighboring Plant P Limitation on P Content in Different Organs of Chinese Fir

Total P concentration (g·kg^−1^) in the whole plant and individual organs of the focal Chinese fir responded to neighbor P treatments in a relatedness-dependent manner. At the whole-plant level (Figure 2h), whole-plant total P was significantly higher under neighbor zero P (P1–P0) than under P1–P1 in the Kin combination (*p* < 0.05); in the Close combination, P1–P0.5 was significantly higher than P1–P1 (*p* < 0.05); and in the Unrelated combination, P1–P0 was significantly higher than both P1–P0.5 and P1–P1 (*p* < 0.05). At the root level (Figure 2i), a significant difference was detected only in the Close combination, where P1–P0 was 56.09% higher than P1–P1 (*p* < 0.05). At the stem level (Figure 2j), P1–P0 was significantly higher than P1–P0.5 and P1–P1 in the Unrelated combination (*p* < 0.05). At the leaf level (Figure 2k), leaf total P in the Unrelated combination was 34.16% higher under P1–P0 than under P1–P1 (*p* < 0.05). The interaction between Phosphorus Supply and Neighbor relatedness (A × B) had a highly significant effect on whole-plant and stem phosphorus contents (*p* < 0.01) (Table 1).

### 2.5. Linear Regression Between Whole-Plant ^13^C Abundance and Whole-Plant P Content of Chinese Fir Under Neighboring Plant P Limitation

**Figure 3:**
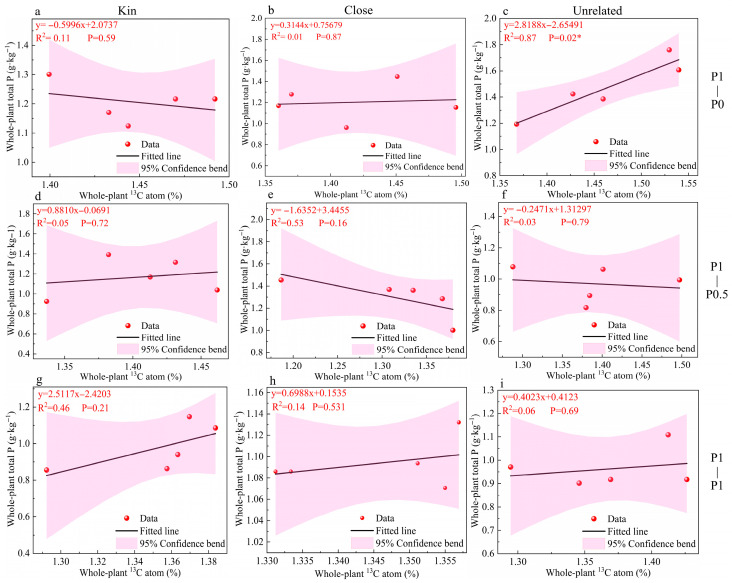
Linear regressions between whole-plant ^^13^C abundance (atom% ^13^C) and whole-plant total P concentration (g·kg^−1^) of the focal Chinese fir under different neighbor P supply gradients and neighbor relatedness combinations. Panels (**a**–**c**) represent the P1–P0 treatment (neighbor zero P), panels (**d**–**f**) represent the P1–P0.5 treatment (neighbor low P), and panels (**g**–**i**) represent the P1–P1 treatment (neighbor P sufficiency). Within each row, columns correspond to the Kin, Close, and Unrelated combinations, respectively. Points represent individual replicates; solid lines indicate fitted linear regressions; shaded areas indicate 95% confidence bands. Regression equations and R^2^ values are shown in each panel. Asterisks denote significant regressions (*p* < 0.05). Kin, Close, and Unrelated indicate that the focal Chinese fir was grown adjacent to kin, closely related, or unrelated neighbor plant, respectively. P1–P0, P1–P0.5, and P1–P1 denote neighbor P supply levels of zero P, low P, and P sufficiency, respectively. Asterisks indicate significant correlations (*p* < 0.05).

As shown in Figure 3a–i, under P1–P0, a significant positive correlation between whole-plant atom% ^^13^C and whole-plant total P was detected only in the Unrelated combination (y = 2.8188x − 2.6549, R^2^ = 0.87, *p* < 0.05) (Figure 3c), whereas the correlations were not significant in the Kin or Close combinations (Figure 3a,b). Under P1–P0.5 and P1–P1, no significant correlations were observed in any relatedness combination (*p* > 0.05) (Figure 3d–i).

### 2.6. Correlation Analysis of Various Indicators of Chinese Fir

**Figure 4:**
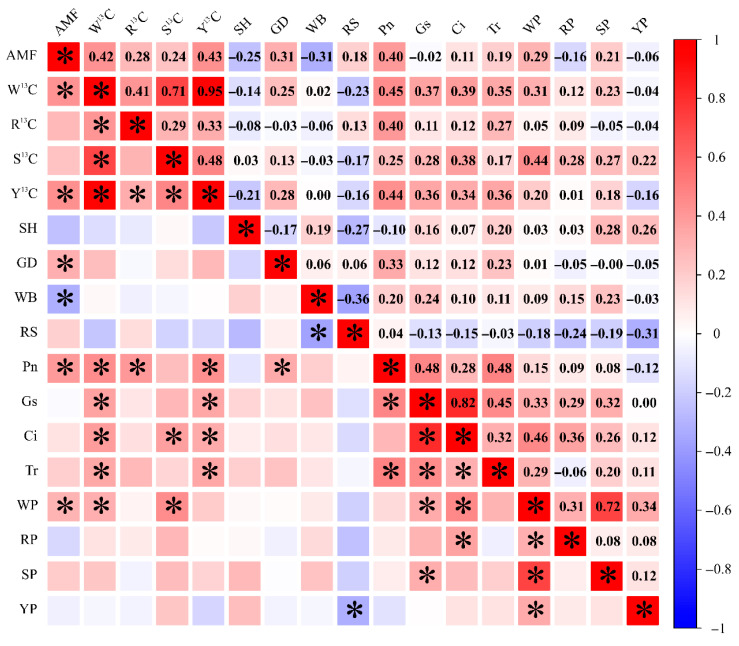
Pearson correlation analysis among mycorrhizal colonization, ^13^C enrichment, growth traits, photosynthetic parameters, and phosphorus (P) concentrations in Chinese fir. Colors indicate the Pearson correlation coefficient (red, positive; blue, negative), and color intensity reflects the magnitude of the correlation. AMF: mycorrhizal colonization rate; W^13^C: whole-plant ^13^C abundance; R^13^C: root ^13^C abundance; S^13^C: stem ^13^C abundance; L^13^C: leaf ^13^C abundance; SH: seedling height increment; GD: ground diameter increment; WB: whole-plant biomass increment; RS: root-to-shoot ratio; P_n_: net photosynthetic rate; G_s_: stomatal conductance; C_i_: intercellular CO_2_ concentration; T_r_: transpiration rate; WP: whole-plant P concentration; RP: root P concentration; SP: stem P concentration; LP: leaf P concentration. Asterisks indicate significant Pearson correlations (*p* < 0.05).

AMF colonization rate was positively correlated with W^13^C (r = 0.42, *p* < 0.05), and also showed significant positive correlations with L^13^C (r = 0.43) and GD (r = 0.31), but a significant negative correlation with WB (r = −0.31, *p* < 0.05). AMF colonization was also positively correlated with Pn (r = 0.40) and WP (r = 0.29) (*p* < 0.05) (Figure 4).

## 3. Discussion

### 3.1. Study on the Response Mechanism of Chinese Fir to Neighboring Plant P Limitation

This study showed that P limitation imposed on neighboring plants significantly increased AMF colonization in the roots of the focal Chinese fir (Figure 2a) and was accompanied by a higher whole-plant P content (Figure 2h) (*p* < 0.05). As a major group of soil symbionts intimately associated with plants, AMF can transmit stress-related cues through hyphal networks and induce adaptive responses in adjacent, connected plants when a host experiences environmental stress [34,35,36]. In the present system, AMF colonization was detected in both the focal Chinese fir (Figure 2a) and neighboring plants with contrasting relatedness (Figure A2a). After ^13^CO_2_ pulse labeling of the focal plant, the ^13^C abundance detected in neighboring plants exceeded their respective natural-abundance baselines (Table A1), a pattern that is consistent with carbon movement within a common mycorrhizal network and thus supports the potential mediating role of AMF in interplant carbon exchange. Similar AMN-enabled interplant communication has been experimentally demonstrated in AMF-connected tomato plants, providing a relevant precedent for network-level signal and resource coupling [37].

Correlation analysis further indicated a significant positive relationship between AMF colonization in Chinese fir roots and whole-plant P content (Figure 4). Tian et al. [38] reported that Chinese fir can cope with low P stress by increasing AMF colonization, enhancing antioxidant enzyme activities, strengthening photosynthesis, and modulating endogenous hormone distribution. In addition, Liu et al. [39] showed that once stress signals were conveyed through arbuscular mycorrhizal networks, the connected “healthy” plant increased AMF colonization and consequently improved stress tolerance. Together with these observations, the present results support an interpretation that AMF may function as a signal carrier of P-limitation cues, triggering an adaptive increase in AMF establishment in the focal plant and thereby strengthening the mycorrhizal P-acquisition pathway under elevated P-limitation risk. Mechanistically, experimental manipulation of host carbon supply in mycorrhizal root organ cultures demonstrated that carbon availability delivered across the mycorrhizal interface can directly trigger fungal nutrient uptake and long-distance transport (shown for N) and is coordinated by shifts in fungal gene expression [40], supporting the view that host carbon flux is a proximal regulator of fungal nutrient delivery and “terms of trade” in AM symbioses.

**Figure A1:**
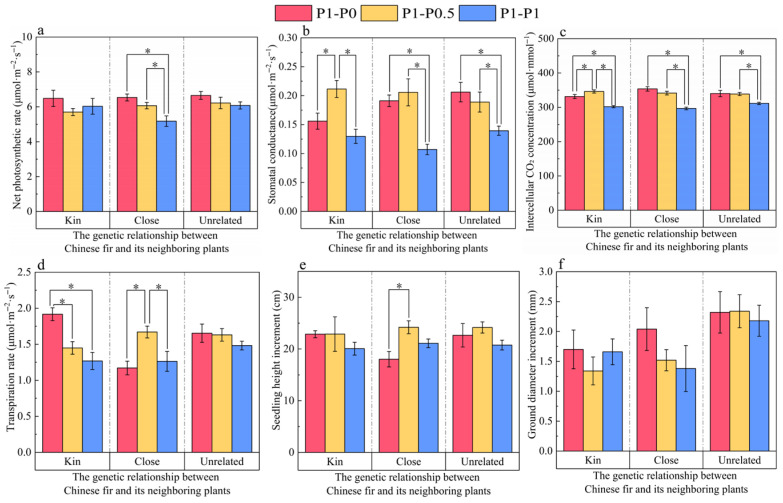
Gas-exchange parameters and growth increments of the focal Chinese fir under different neighbor P-supply gradients and relatedness combinations. (**a**) Net photosynthetic rate (Pn), (**b**) stomatal conductance (Gs), (**c**) intercellular CO_2_ concentration (Ci), (**d**) transpiration rate (Tr), (**e**) seedling height increment (SH), and (**f**) ground diameter increment (GD). Kin, Close, and Unrelated indicate that the focal Chinese fir was grown adjacent to a kin, closely related, or unrelated neighbor plant, respectively. P1–P0, P1–P0.5, and P1–P1 represent the zero P, low P, and sufficient P treatments applied to the neighbor plant, respectively. Data are presented as mean ± SE (*n* = 5). Asterisks indicate significant differences (*p* < 0.05).

In parallel, neighbor P limitation was associated with higher whole-plant ^13^C enrichment of the focal Chinese fir, particularly under the P1–P0 condition (Figure 2d), and AMF colonization was positively correlated with whole-plant ^13^C enrichment (Figure 4). Although gas-exchange traits (Figure A1a–d) may vary among combinations, the concurrent increase in ^13^C enrichment and colonization supports a carbon–nutrient exchange framework. Photosynthetically fixed carbon is not only the fundamental substrate for plant growth but also the primary currency sustaining the reciprocal trade of “photosynthetic carbon and mineral nutrients” between AMF and host plants [41]. Under stress, increased mineral nutrient transfer from AMF to hosts is often coupled with increased carbon acquisition by AMF to maintain fungal metabolism and symbiotic stability [42]. In line with the “biological market” concept, plants can reward more cooperative fungal partners with more carbon, stabilizing reciprocal exchange [43]. Consistently, Hong et al. [44] demonstrated, using ^13^CO_2_ labeling, that under low P conditions, AMF increased the proportion of host carbon allocated to colonized roots and fungal hyphae, thereby promoting fungal growth and helping hosts cope with P limitation. Therefore, it is proposed that when neighboring plants experience P limitation, Chinese fir may increase carbon assimilation and/or allocate more photosynthate to AMF as a higher “C reward”, which could promote P return through the mycorrhizal pathway and mitigate potential P-limitation risk.

### 3.2. Study on the Regulation of Kin Recognition on the Response Strategy of Chinese Fir to Neighboring Plant P Limitation

Kin recognition is widely considered an important regulator of intra- and interspecific competitive intensity and can ultimately shape plant growth strategies and resource allocation [45]. In the present study, response patterns of the focal Chinese fir differed among the three neighbor-relatedness combinations under neighbor P limitation. Specifically, under the neighbor zero P treatment, a significant increase in root ^13^C enrichment was detected only when the focal plant grew adjacent to a kin neighbor (Figure 2e), whereas plants grown with an unrelated neighbor exhibited significant increases in stem ^13^C enrichment (Figure 2f) and stem P concentration (Figure 2j). These organ-specific shifts are consistent with the notion that plants can adjust competitive investment depending on the perceived “self–non-self” context, thereby reducing wasteful intraspecific competition while enhancing performance when facing unrelated-kin competitors [46,47]. Moreover, accumulating evidence suggests that AMF and their hyphal networks can participate in kin-related interactions by transmitting cues and/or mediating resource exchange, potentially providing measurable benefits under certain neighbor contexts [27].

Consistent with this view, growth and allocation traits of the focal Chinese fir responded most clearly in the unrelated combination. Under the neighbor low P treatment, whole-plant biomass increment was significantly higher than that under neighbor P sufficiency (Figure 2b), while under the neighbor zero P treatment, the root-to-shoot ratio was significantly reduced relative to neighbor P sufficiency (Figure 2c). In contrast, neither biomass increment nor root-to-shoot ratio differed significantly among neighbor P levels in the kin and close-kin combinations (Figure 2b,c). One plausible interpretation is that, when an unrelated neighbor experiences P limitation, the focal Chinese fir may prioritize aboveground investment to maintain competitive advantage, a pattern that is conceptually compatible with competition-induced allocation shifts (e.g., shade-avoidance–type responses) mediated by neighbor cues [48].

In addition, the relationship between whole-plant ^13^C enrichment and whole-plant P concentration depended on neighbor relatedness under P limitation (Figure 3). In the unrelated combination, the positive association between ^13^C enrichment and P concentration became stronger under neighbor zero P and reached significance (Figure 3; *p* < 0.05), whereas in the kin combination, the fitted relationship under neighbor zero P shifted toward a negative trend and did not show a significant correlation. Although correlation patterns alone cannot establish directionality, these contrasting slopes are consistent with a relatedness-dependent “trade configuration” within the Chinese fir–AMF network. In particular, when facing an unrelated P-limited neighbor, increased C input (reflected by higher ^13^C enrichment) appears to coincide with greater P accumulation in the focal plant, suggesting a more self-benefiting exchange outcome. By contrast, when the neighbor is kin, increased C input by the focal plant does not translate into higher P accumulation in its own tissues, which is compatible with the possibility that network-level resource redistribution favors kin-associated sinks. This interpretation aligns with prior findings that plant kin context can enhance investment in the mycorrhizal network and modify the realized benefits of network association [49].

**Figure A2:**
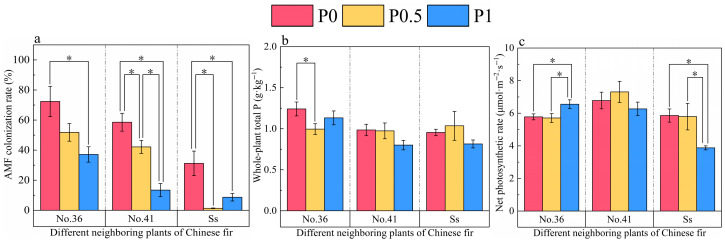
Responses of neighboring plants to different P-supply treatments. (**a**) Root AMF colonization rate, (**b**) whole-plant P concentration, and (**c**) net photosynthetic rate (Pn). No. 36 and No. 41 indicate the kin neighbor (Chinese fir clone No. 36) and the close-kin neighbor (Chinese fir clone No. 41), respectively, whereas Ss indicates the unrelated neighbor plant (*Schima superba*). P0, P0.5, and P1 represent the zero P, low P, and sufficient P treatments, respectively. Data are presented as mean ± SE (*n* = 5). Asterisks indicate significant differences (*p* < 0.05).

To further evaluate whether kin neighbors under zero P (P0) conditions could receive network-level support, P concentration and photosynthetic performance were measured in neighboring plants. Under the P0 treatment, the kin neighbor showed a significant increase in whole-plant P concentration (Figure A2b; *p* < 0.05) but a significant decrease in net photosynthetic rate (Figure A2c; *p* < 0.05). In a strict one-to-one exchange framework, reduced host carbon supply is often expected to weaken mycorrhiza-mediated nutrient delivery [50,51]. However, in multi-host networks, AMF resource allocation may be constrained more strongly by the net carbon economy of the network, and fungi may compensate for carbon deficits of stressed hosts by acquiring additional C reward from adjacent healthier hosts, thereby maintaining nutrient delivery to the stressed end [52,53]. In addition, previous studies have shown that when biotic stress markedly reduces or nearly interrupts host carbon supply to AMF, nutrient delivery to the stressed host can still be maintained if neighboring healthy plants subsidize the fungal partner with carbon [54]. A possible explanation, consistent with the studies mentioned above, may be that under P limitation, AMF-mediated P delivery to kin neighbors could be maintained even when the neighbors’ photosynthetic capacity declines. This may be because nutrient allocation in multi-host networks can be constrained by network-level carbon balance. Based on the ^13^C enrichment patterns observed in the focal plants, we suggest that P-sufficient focal Chinese fir might provide a relatively higher C reward to AMF and thereby potentially partially subsidize the carbon deficit of the P-limited kin neighbor. This mechanism could help sustain network functioning, but it still needs to be verified by quantifying carbon transfer and nutrient fluxes within mycorrhizal networks. Future studies incorporating ^13^C measurements from neighbor tissues and/or AMF-related parameters would further strengthen the evidence for carbon movement at the network level. Collectively, these findings suggest that neighbor relatedness can act as an important factor that reconfigures common mycorrhizal network-mediated carbon–phosphorus exchange under heterogeneous P supply. Future work integrating multi-omics approaches is warranted to identify the key genes and signaling molecules underpinning kin recognition, AMF signal transmission, and carbon–phosphorus allocation, and to clarify the associated regulatory pathways.

**Figure 5:**
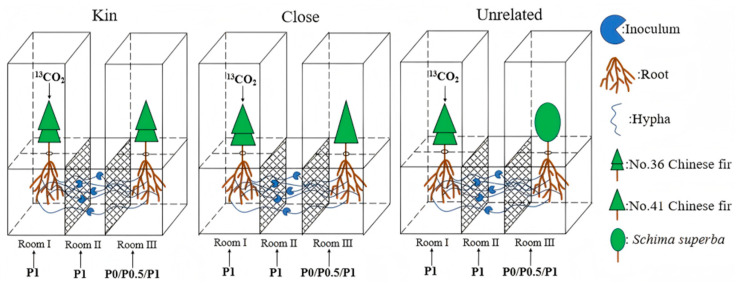
Schematic diagram of the experimental apparatus for the “Chinese fir–AMF–P-limited neighbor” symbiotic network system, with 5 biological replicates per treatment.

Despite the clear treatment-dependent patterns observed in this controlled system, the present study provides initial evidence that AMF-mediated networks are involved in Chinese fir responses to neighbor P limitation and that kin recognition may modulate this response, several constraints inherent to enclosed ^13^CO_2_ pulse labeling should be acknowledged. To prevent label leakage and cross-contamination among Rooms during targeted ^13^CO_2_ labeling (Figure 5), the experiment could not be fully randomized across multiple independent Rooms. Therefore, although biological replication was implemented at the pot (apparatus) level (*n* = 5), Room-specific heterogeneity (e.g., microclimate, airflow, and gas mixing) may partially confound treatment effects. In this context, while pot-level replication supports comparisons among treatments, future studies incorporating independent Room replication and stronger Room-level randomization would help further rule out potential Room-related confounding.

## 4. Materials and Methods

An indoor pot experiment simulating heterogeneous P supply was conducted to examine how P limitation alters the reciprocal exchange of “photosynthetic carbon and mineral phosphorus” between Chinese fir and AMF when a focal plant grows adjacent to neighbors with different degrees of relatedness. Clonal seedlings of Chinese fir No. 36 were used as the focal plant, and Chinese fir No. 36, Chinese fir No. 41, and *Schima superba* were used as neighboring plants. Three two-plant combinations were established to represent contrasting relatedness: a kin pair (No. 36 + No. 36), a close-kin pair (No. 36 + No. 41), and an unrelated-kin pair (No. 36 + *S. superba*). A shared root-zone (common Room) was inoculated with *Funneliformis mosseae* to establish a mycorrhizal network connecting the two plants. The neighboring plant was subjected to a gradient of P limitation (sufficient P, low P, and zero P), whereas the focal No. 36 plant received ^13^CO_2_ pulse labeling to generate a symbiotic network in which No. 36 served as the ^13^C donor within a “Chinese fir–AMF–P-limited neighbor” system.

### 4.1. Experimental Materials

One-year-old clonal seedlings of Chinese fir No. 36 and No. 41, originating from distinct geographic–ecological types within the core production region and previously selected by the Chinese Fir Engineering Technology Research Center of the National Forestry and Grassland Administration of China, were used as experimental materials. In addition, one-year-old seedlings of *Schima superba* (a common native broadleaf tree species in subtropical China) were also included, as this species is often used for interplanting under Chinese fir forests in practice, which is consistent with the actual planting pattern of subtropical Chinese fir plantations. The No. 36 and No. 41 clones differ substantially in P-foraging strategies under P limitation: No. 41 adopts an “active” strategy for adaptation to low P conditions, whereas No. 36 has the capacity to initiate a “passive” strategy, and the relatedness between the two clones is close kin [55]. All seedlings were healthy, free of pests and diseases, uniform in size, and well developed. Initial seedling height and ground diameter were expressed as the mean ± standard error (Table 2). Washed river sand (total P concentration: 0.09 ± 0.01 mg kg^−1^, by the molybdenum–antimony anti-spectrophotometric method) was used as the potting substrate. Prior to planting, the sand was packed into sterilization bags made of high-density polyethylene film (Beijing Xinminghong Plastic Co., Ltd., Beijing, China) and autoclaved using a vertical steam sterilizer (Shanghai Shen’an Medical Instrument Factory, Shanghai, China) at 121 °C (0.1–0.2 MPa) for 30 min. The AMF inoculum (*Funneliformis mosseae*) was provided by the Arbuscular Mycorrhizal Fungi Germplasm Resource Bank, Institute of Plant Nutrition and Resources, Beijing Academy of Agriculture and Forestry Sciences (Beijing, China). The inoculum was propagated using *Sorghum bicolor* as a host plant; after 3 months of pot culture, the inoculum was harvested, including the entire substrate, colonized root fragments, spores, and hyphae, and stored at 4 °C until use.

### 4.2. Experimental Design

#### 4.2.1. Pot Experiment

The experiment followed a two-factor completely randomized factorial design. The first factor was neighbor type, including same clone No. 36–No. 36 (kin), different clones within the same species No. 36–No. 41 (close), and a heterospecific neighbor No. 36–*Schima superba* (unrelated), and the second factor was the P-supply gradient for the neighbor, including 1.0 mmol·L^−1^ KH_2_PO_4_ (P1), 0.5 mmol·L^−1^ KH_2_PO_4_ (P0.5), and 0 mmol·L^−1^ KH_2_PO_4_ (P0). Thus, there were 9 treatments (three neighbor types × three P gradients in room III). Each treatment had 5 independent replicates, resulting in 45 independent experiment units in total. We used self-designed “Chinese fir–AMF–P-limited neighbor” pot apparatuses (i.e., experiment units; Figure 5) to conduct the pot-based simulation experiment.

The apparatus comprised three rooms (I, II, and III) separated by 25-μm nylon meshes (China, 600 mesh, single layer), which allow hyphal passage but prevent root penetration, thereby avoiding direct root contact between plants in adjacent rooms [56]. No root penetration across the meshes was observed during the experiment by periodic inspection. One target Chinese fir seedling (No. 36) was planted in room I, and the neighbor plant (Chinese fir No. 36, Chinese fir No. 41, or *S. superba*) was planted in room III. Room II was used for AMF inoculation: 200 g of AMF inoculum was thoroughly mixed with the potting substrate in this room (washed and sterilized river sand) to inoculate *Funneliformis mosseae*, establish mycorrhizal symbiosis, and enable hyphal connections extending across the mesh. Based on the P status of subtropical forest soils in China [57], rooms I and II of all apparatuses received sufficient P supply (1.0 mmol·L^−1^ KH_2_PO_4_, P1), whereas room III received three P treatments (P1, P0.5, and P0), thereby generating three neighbor-P gradients (P1–P1), (P1–P0.5), and (P1–P0) (rooms I/II–room III) and forming a “Chinese fir–AMF–P-limited neighbor” symbiotic network system. In the low P and zero P treatments, K^+^ was balanced with KCl, and all other nutrients were supplied using a modified quarter-strength incomplete Hoagland solution following Wu et al. [58]. During the treatment period, nutrient solutions corresponding to the target P levels were added separately to each room every 7 d (Figure 5). Prior to planting, the apparatuses were disinfected with 0.1 g·L^−1^ KMnO_4_ for 20 min, rinsed with sterile water, and air-dried.

Seedling roots were surface-sterilized with 0.05% NaClO for 15 s and then rinsed five times with sterile water before transplanting into the apparatus. All seedlings were planted in February 2024 and allowed to acclimate for 1 month before treatments began, and the treatment period lasted for 6 months with destructive harvest of all seedlings conducted in September 2024; roots, stems, and leaves were collected separately for subsequent analysis.

#### 4.2.2. ^13^C Isotope Pulse-Labeling Design

Based on the pot experiment described above (Figure 5), with a ^13^CO_2_ pulse-labeling assay in a greenhouse (temperature 26–30 °C, relative humidity ~75%, and a 14 h·d^−1^ photoperiod). The ^13^CO_2_ pulse labeling was conducted in room I, where the focal Chinese fir clone No. 36 was grown. Pulse labeling started 40 days before harvest and was performed once every 10 days for a total of four pulses. Each pulse was applied between 09:00 and 12:00. Following Li et al. [59], a beaker was placed in the labeling room and 0.10 g Na_2_^13^CO_3_ (99 atom% ^13^C; ICON, St. Charles, MO, USA; Shanghai Saifei Biotechnology Co., Ltd., Shanghai, China) was reacted with 2.0 mol·L^−1^ HCl to generate ^13^CO_2_. The released ^13^CO_2_ was assimilated by the focal plant via photosynthesis, thereby labeling newly fixed carbon. To prevent excessive temperature increases inside the labeling room during the reaction, ice packs were added as needed until the Na_2_^13^CO_3_ reaction was complete. To avoid ^13^C contamination of unlabeled seedlings, labeled and control treatments were spaced at least 15 m apart.

### 4.3. Experimental Methods

#### 4.3.1. Determination of AMF Root Colonization Intensity

After destructive harvest, newly formed roots were randomly sampled from each seedling and cut into 30–50 root segments (1 cm in length). Root segments were fixed in FAA solution for 5 h, rinsed with distilled water, and stained using the trypan blue method [60]. The colonization level of each individual root segment was recorded, and AMF root colonization (%) for each seedling was calculated according to the following equation:

AMF root colonization (%) = (∑ M × *n*)/N

where M is the colonization class (colonization percentage) of an individual root segment, *n* is the number of root segments with the same colonization class, and N is the total number of root segments assessed per seedling.

#### 4.3.2. Determination of ^13^C Abundance

δ^13^C abundance was determined using an elemental analyzer–isotope ratio mass spectrometry system (EA-IRMS), consisting of an elemental analyzer (vario ISOTOPE cube, Elementar, Hanau, Germany) and an isotope ratio mass spectrometer (IRMS Isoprime 100, Elementar, Manchester, UK). The carbon isotope composition (δ^13^C) was calculated by comparing the isotope ratio of the sample with that of the reference standard [61,62]:

δ^13^C (‰) = (Rsa/RPDB − 1) × 1000

where Rsa is the ^13^C/^12^C ratio of the sample (Rsa = (^13^C/^12^Csa), and RPDB is the ^13^C/^12^C ratio of the standard (RPDB = ^13^C/^12^C = 0.0112372). For labeled plants, ^13^C abundance is commonly expressed as atom% (^13^C). The conversion from δ^13^C (‰) to atom% was performed using the following equation:

Atom(%) = [(δ^13^C + 1000) × RPDB]/{[(δ^13^C + 1000) × RPDB] + 1000 × 100}

To exclude the influence of natural ^^13^C abundance, atom% (^13^C) was measured in unlabeled seedlings (Chinese fir No. 36, Chinese fir No. 41, and *Schima superba*). The mean natural abundance (atom% ^13^C) in roots, stems, and leaves was 1.08%. After ^13^CO_2_ pulse labeling, atom% ^13^C in roots, stems, and leaves of all seedlings was higher than the corresponding natural abundance.

#### 4.3.3. Determination of Growth Indicators

Before transplanting, 15 seedlings of Chinese fir No. 36, Chinese fir No. 41, and *Schima superba* were randomly selected, separated into roots, stems, and leaves, and oven-treated at 105 °C for 30 min to deactivate enzymes. The temperature was then reduced to 75 °C and samples were dried to constant mass. Dry weights of roots, stems, and leaves were recorded as the initial biomass of each organ. At the time of transplanting, seedling height and ground diameter were measured for each seedling and recorded as initial values. At the end of the experiment, following destructive harvest, seedling height and ground diameter were measured again and recorded as final values. Growth performance was evaluated as the increment in seedling height and ground diameter (final minus initial).

After destructive harvest, roots, stems, and leaves were separated and their dry weights were determined as the final organ biomass. Biomass change was assessed as the increment in whole-plant biomass (final minus initial). The root-to-shoot ratio was calculated as root biomass divided by aboveground biomass.

#### 4.3.4. Determination of Physiological Indicators

Before destructive harvest, photosynthetic parameters were measured on a clear day between 08:30 and 11:00 using a LI-6400 portable photosynthesis system (LI-COR Biosciences, Lincoln, NE, USA). The measurement conditions were set as follows: photosynthetically active radiation intensity 1000 μmol·m^−2^·s^−1^, reference CO_2_ concentration 400 μmol·m^−2^·s^−1^, leaf chamber gas flow rate 500 μmol·s^−1^, and leaf chamber temperature was naturally controlled with ambient temperature. Measurements were conducted on healthy, fully expanded mature leaves from newly formed shoots of each seedling. For phosphorus determination, oven-dried samples from each plant organ were digested using a concentrated HNO_3_–HClO_4_ acid digestion procedure, and total P concentration (g·kg^−1^) in the digest was quantified using the molybdenum–antimony anti-spectrophotometric colorimetric method [63].

### 4.4. Data Statistics and Analysis

Two-way analysis of variance (two-way ANOVA) was used to test the main effects of neighbor relatedness, P-supply gradient, and their interaction on AMF colonization, seedling growth, and the dynamics of ^13^C enrichment and phosphorus (P) concentration in tissues of the target Chinese fir. Post hoc multiple comparisons were performed to test treatment differences among P-supply gradients within each neighbor-relatedness level using the least significant difference (LSD) test (*p* < 0.05). Data were tested for normality and homogeneity of variance prior to ANOVA, and were transformed when necessary to meet ANOVA assumptions. Linear regression analyses were conducted to examine relationships between whole-plant ^13^C abundance (atom% ^13^C) and whole-plant total P concentration (g·kg^−1^) of the focal Chinese fir under different neighbor P-supply gradients and neighbor relatedness combinations. Pearson correlation analysis was used to evaluate associations among mycorrhizal colonization, ^13^C enrichment, growth traits, photosynthetic parameters, and P concentrations in Chinese fir. All data are presented as mean ± standard error (SE). Statistical analyses were performed using SPSS 26.0 (IBM, Corp., Armonk, NY, USA). Figures were generated using Origin 2022 (OriginLab, Northampton, MA, USA).

## 5. Conclusions

When neighboring plants experienced phosphorus limitation, Chinese fir showed increased AMF colonization in roots and enhanced leaf photosynthetic performance, accompanied by greater accumulation of photosynthetically derived carbon and higher phosphorus uptake. The allocation pattern depended on neighbor relatedness. When the neighbor was a conspecific kin individual, Chinese fir exhibited increased belowground ^13^C enrichment, which may partially compensate for the AMF carbon demand (i.e., the C reward) for the P-limited kin neighbor. In contrast, when the neighbor was a heterospecific unrelated-kin individual, Chinese fir exhibited increased stem ^13^C enrichment and stem phosphorus accumulation, coinciding with enhanced aboveground growth. Overall, this study provides initial evidence that AMF-mediated networks are involved in Chinese fir responses to neighbor P limitation and suggests that kin recognition may modulate this response. These findings have practical implications for forestry management, particularly for the establishment and tending of Chinese fir plantations. Understanding carbon and phosphorus regulation via AMF networks and kin recognition could help optimize mixed planting patterns, neighbor configuration, and phosphorus fertilization strategies, thereby improving resource use efficiency and the sustainability of Chinese fir production in practice.

Future studies can integrate multi-omics technologies to identify the key genes and signaling molecules mediating kin recognition, AMF signal transduction, and the regulation of carbon and phosphorus allocation, and to further clarify their underlying molecular regulatory pathways. Additionally, independent Room replication (i.e., more than one independent Room per treatment) should be incorporated whenever feasible. Where the number of available Rooms is limited, repeating the experiment across multiple independent runs (temporal replication) and applying treatment–Room rotation between runs may help reduce potential Room effects. In the meantime, standardized randomization and periodic rotation of pot positions within each Room, together with continuous monitoring of environmental variables, would further improve experimental rigor and enhance reproducibility and generalizability, thereby enabling a more comprehensive assessment of AMF-network functional dynamics under heterogeneous P supply and their dependence on neighbor relatedness.

## Figures and Tables

**Table 1:** Two-way ANOVA analysis with Phosphorus supply and Neighbor relatedness on AMF colonization, seedling growth, and the dynamics of ^13^C enrichment and P concentration in tissues of the focal Chinese fir.

	Phosphorus Supply (A)	Neighbor Relatedness (B)	A × B
AMF	**	ns	ns
W^13^C	**	*	ns
R^13^C	**	*	ns
S^13^C	**	ns	ns
L^13^C	**	ns	ns
SH	ns	ns	ns
GD	ns	**	ns
WB	ns	ns	ns
RS	ns	ns	ns
Pn	**	ns	ns
Gs	**	ns	ns
Ci	**	ns	*
Tr	**	*	**
WP	**	ns	**
RP	ns	ns	ns
SP	**	ns	**
LP	ns	ns	ns

Note: AMF: mycorrhizal colonization rate; W^13^C: whole-plant ^13^C abundance; R^13^C: root ^13^C abundance; S^13^C: stem ^13^C abundance; L^13^C: leaf ^13^C abundance; SH: seedling height increment; GD: ground diameter increment; WB: whole-plant biomass increment; RS: root-to-shoot ratio; Pn: net photosynthetic rate; Gs: stomatal conductance; Ci: intercellular CO_2_ concentration; Tr: transpiration rate; WP: whole-plant P concentration; RP: root P concentration; SP: stem P concentration; LP: leaf P concentration. ‘*’ and ‘**’ indicate significant differences at *p* < 0.05, and *p* < 0.01, respectively, and ‘ns’ represents no significant difference.

**Table 2:** The initial value of seedling height and ground diameter of Chinese fir and *Schima superba.*

Experimental Seedlings	Initial Seedling Height (cm)	Initial Ground Diameter (mm)
Chinese fir No. 36	21.91 ± 0.30	3.08 ± 0.06
Chinese fir No. 41	23.93 ± 0.84	3.08 ± 0.14
*Schima superba*	20.17 ± 0.65	2.93 ± 0.11

## Data Availability

The dataset supporting this study is included in the article. Further inquiries can be directed to the corresponding author.

## Abbreviations

The following abbreviations are used in this manuscript:

Kin	The focal Chinese fir was grown adjacent to a kin neighbor plant
Close	The focal Chinese fir was grown adjacent to a close neighbor plant
Unrelated	The focal Chinese fir was grown adjacent to an unrelated neighbor plant
P1–P0	Neighbor P supply levels of zero P
P1–P0.5	Neighbor P supply levels of low P
P1–P1	Neighbor P supply levels of P sufficiency
AMF	Arbuscular mycorrhizal fungi
W^13^C	Whole-plant ^13^C abundance
R^13^C	Root ^13^C abundance
S^13^C	Stem ^13^C abundance
L^13^C	Leaf ^13^C abundance
SH	Seedling height increment
GD	Ground diameter increment
WB	Whole-plant biomass increment
RS	Root-to-shoot ratio
Pn	Net photosynthetic rate
Gs	stomatal conductance
Ci	Intercellular CO_2_ concentration
Tr	Transpiration rate
WP	Whole-plant P concentration
RP	Root P concentration
SP	Stem P concentration
LP	Leaf P concentration

## Appendix A

**Table A1:** Natural and measured whole-plant atom% ^13^C of neighboring plants under different phosphorus supply treatments.

Neighboring Chinese Fir Plant	Phosphorus Supply Treatment	Natural Whole-Plant ^13^C Atom (%)	Determined Whole-Plant ^13^C Atom (%)
No. 36	P0	1.08	1.10
	P0.5	1.08	1.09
	P1	1.08	1.10
No. 41	P0	1.08	1.14
	P0.5	1.08	1.09
	P1	1.08	1.10
Ss	P0	1.08	1.11
	P0.5	1.08	1.10
	P1	1.08	1.09

Note: No. 36 and No. 41 indicate the kin neighbor (Chinese fir clone No. 36) and the close-kin neighbor (Chinese fir clone No. 41), respectively, whereas Ss indicates the unrelated neighbor plant (*Schima superba*). P0, P0.5, and P1 represent the zero P, low P, and sufficient P treatments, respectively.
